# Substance of a Clinical Lecture on a Case of Embolon of the Femoral Artery, Etc.

**Published:** 1862-12

**Authors:** 


### SUBSTANCE OF A CLINICAL LECTURE ON A CASE

### OF EMBOLON OF THE FEMORAL ARTERY PRODUCING GANGRENE OF THE FOOT—FATAL RESULT.

**Under the care of Dr. Pitman and Mr. Prescott Hewett.**

James M——, aged fifty-six, was admitted in November, 1861, under the care of Dr. Pitman. The patient was just convalescent from a severe attack of dropsy connected with heart-disease; and he was going about the ward up to the 17th of December, when he was suddenly seized with violent pain in the left groin, "exactly as if he had been shot." From that moment the limb became numb, cold, and quite powerless: "it felt as if it was dead;" and he was obliged at once to take to his bed again. All that afternoon and night he suffered intensely; and when seen by Mr. Hewett, at Dr. Pitman's request, he was still complaining of great pain in the whole of the limb, but especially in the groin. The limb was colder than the rest of the body; and there were blotches of discoloration spreading over the toes and back of the foot, with a slight œdema, and considerable redness extending half-way up the leg. A careful examination of the groin, at the exact point where the pain had been so suddenly experienced the day before, showed that the femoral artery was plugged. Reduced as the patient was from long-continued suffering, the size and exact limits of the plug could be distinctly made out. It began at about two inches below Poupart's ligament, and extended for three-fourths of an inch down the course of the vessel. At this spot it was quite hard, round, and rolled under the finger; pulsation was felt above, but not below, the plug; no pulsation was present in any of the arteries of the leg or foot. The general aspect of the patient was unhealthy; the skin of yellow tinge; countenance sunken, anxious, and worn; pulse intermittent and very feeble. The heart's action was irregular and feeble; but there was no valvular disease. The diagnosis arrived at was blocking up of the femoral artery by a plug of fibrin washed from the heart. The limb was wrapped in cotton-wool, compound soap liniment was rubbed in the groin to ease the pain, morphine was given, and stimulants freely. The gangrene advanced slowly about the foot; the toes became shrivelled, black, dry, and nummified; and the œdema and redness increased. The man became weaker and weaker, and died on the 23rd of January, 1862. A few days before his death the gangrene ceased, after involving two-thirds of the foot. On a consultation, considering the man's low typhoid condition, it was decided not to attempt any operation.

*Autopsy.*—The plug in the artery measured three-quarters of an inch, and was situated in the upper part of the superficial femoral; and, exactly corresponding to the seat of obstruction, the parts around were thickened, and the vessel was firmly adherent to the sheath and to the vein. This plug was throughout adherent, and was hard, rusty on its surface, fawn-colored, deep-seated, and like the fibrinous layers of an old aneurism. The heart was large and flabby; there was no endocarditis nor disease of the valves, but entangled among the fleshy columns of the left ventricle, and filling up and projecting beyond the interspaces, where several large fibrinous masses, for the most part of a fawn color, very firm, and evidently of long standing; and a similar mass, of large size, was also found blocking up the left auricular appendix and projecting into the cavity of the auricle itself, where it was pendulous. Besides these there were also (especially in the right ventricle) some loose coagula of recent formation. The large vessels were healthy. The lungs were inflamed at their lower part. At the upper and lower parts of the spleen were two very large fibrinous concretions, of a deep-yellow color and firm in texture; and the arteries leading to them were found blocked up with fibrin. All the other viscera were healthy.

In commenting upon the case, Mr. Hewett remarked that it afforded for consideration some important points in surgery. First, as to the diagnosis. What led to the conclusion that the case was one of embolism? "The gangrene of the foot and the condition of the leg showed that there was obstruction of the arterial system in some part of the limb. Then came the question as to the precise locality of the obstruction. This man, you will remember, had "felt as if he had been shot in the groin." Well, that led at once to the examination of the femoral artery at this spot; and there the vessel was found blocked up. But what caused the obstruction? Was it an embolus? The sudden shooting pain in the groin, the immediate loss of sensation in the limb, the complete obstruction of the vessel, the limited extent of the plug, and its precise locality—about the giving-off of the deep femoral,—all tended to prove that the plug had not originated in the artery itself, but had come from a distance. Whence came the plug? From the left cavities of the heart, in all probability. To this, however, it may be objected that there was nothing to prove the existence of anything like vegetations on the surface of the valves. True; but there may be some fibrinous coagula entangled in the meshes of the fleshy fibres of the heart, and one of these coagula may have been washed from its place, and swept along the arterial current until it was suddenly stopped by getting into a vessel through which it was too large to pass. And, moreover, even supposing that vegetations had existed on the valves, a vessel of the size of the femoral could hardly have been blocked up by one of these vegetations set loose. The portions of such vegetations when washed off from the endocardium are generally of too small a size to plug the main artery of a limb; but they pass into the lesser branches, and, for the most part, lead to mischief about the brain, the spleen, the kidneys, or the liver."

That this reasoning was correct post-mortem inspection proved. A reference was made to cases recorded by M. Nelaton and Mr. Sibley: in them, as in the foregoing case, the embolism was the result of a fibrinous clot, and not arising from vegetations.

Mr. Hewett then entered into the question whether the case was one of arteritis, and not of embolism; and he showed that it could not be the former, chiefly for the reasons that the man lived thirty-seven days after the commencement of the attack (hence the adhesion of the plug to the artery), and that the inflammation did not precede but followed the plugging of the artery; and the condition of the spleen after death further proved the case to be one of embolism.

The question of amputation in gangrene connected with embolism he thought an important one. In the present instance it was set aside by the patient's general condition, which was so thoroughly hopeless that all thought of operating was abandoned, even from the beginning. Had the general health been otherwise, amputation might have been performed with good prospects of success. In a case of embolism with obstruction high up in the limb he thought amputation quite useless until after the setting up of the line of demarcation, without which it is impossible to say what parts may or may not become involved in the gangrene. But in a case of rapidly advancing gangrene, in which the patient has but too evidently only a few hours to live unless something is done, and that without delay, it would be right to amputate at once, provided the plug in the artery be at a sufficient distance from the trunk to admit of removing the limb above the seat of obstruction.

In conclusion, Mr. Hewett referred to the treatment of aneurism by breaking up the coagula, which had been proposed by Mr. Ferguson. This treatment he considered, in fact, embolism artificially produced; and if resorted to in aneurisms connected with the current of blood proceeding to the head, it may, there is no doubt, lead to disastrous results —results the very same as those described in Dr. Kirk's paper. Hemiplegia, doubtless due to embolism, followed the breaking up of the coagula in an aneurism of the subclavian artery in one of Mr. Ferguson's cases. Last year another was mentioned by Mr. Teale, of Leeds; and a third case, for the reference to which he (Mr. Hewett) was indebted to his friend Mr. Ernest Hart, occurred in the practice of Professor Esmarsh, of Vienna. In this last case, post-mortem inspection proved the truth of the supposed cause of the head symptoms.—*London Lancet.*

